# Prevention of dementia: Role of vascular risk factors and cerebral emboli

**Published:** 2009-01

**Authors:** Nitin Purandare

**Affiliations:** Psychiatry Research Group, The University of Manchester, University Place, Manchester, UK

**Keywords:** Alzheimer’s disease, vascular dementia, prevention, hypertension, stroke, emboli

## Abstract

Dementia is a major health problem worldwide and the number of people affected is expected to rise considerably, especially in developing countries like India. Vascular risk factors are involved in causation of both vascular dementia and Alzheimer’s disease (AD), account for 90% of all dementias. A selective review of the literature was conducted to summarize the current evidence from clinical studies to examine the role of vascular risk factors in prevention of dementia. Epidemiological evidence suggests that control of vascular risk factors may prevent, or at least delay, the onset of dementia. This finding is supported to some extent by randomized controlled trial evidence for treatment of hypertension but not for other risk factors. However, a number of methodological issues need addressing. There is a need for a randomized controlled trials (RCT) targeting multiple vascular risk factors in patients at increased risk of dementia; i.e., those with mild cognitive impairment. The research should also explore novel risk factors and mechanisms of vascular brain damage. For example, asymptomatic spontaneous cerebral emboli have been shown to be more frequent and associated with a more rapid progression of dementia in both AD and vascular dementia.

## INTRODUCTION

Dementia affects over 24 million people worldwide.[1] In India, the prevalence of dementia in those over the age of 65 years is 1-3%[2] and the prevalence may be higher in urban compared to rural areas. The prevalence of dementia is expected to rise over next three to four decades with biggest increase in developing countries like India.[1] The precise economic and social burden of dementia in India remains unknown but there is some evidence to suggest higher economic cost and carer strain.[3] Dementia is a chronic progressive condition with no cure and delaying its onset could have a big impact on its prevalence. If the onset of dementia could be delayed by about five years then its prevalence could almost be halved.[4]

Alzheimer’s disease (AD) and vascular dementia (VaD) account for about 90% of all dementias with evidence of considerable overlap between the two and “mixed” dementia is common.[2,5] The Nun study showed the additive effect of cerebrovascular and neurodegenerative pathologies in producing clinical dementia. A fewer neuropathological lesions of AD were required for the clinical manifestation of dementia in those who had infarcts in basal ganglia, thalamus or deep white matter.[6] This selective review focuses on the potential role of vascular risk factors, specifically evidence from clinical studies, in the prevention of AD and VaD.

## VASCULAR RISK FACTORS

Hypertension, hypercholesterolaemia, elevated homocysteine, diabetes, heart disease, smoking, and carotid artery disease are known risk factors for dementia, AD, and VaD.

### Hypertension

A number of cross-sectional and epidemiological studies have shown than hypertension in midlife is a risk factor for dementia in old age. For example, Honolulu Asia Aging Study (HAAS) followed 3731 Japanese American men for over 14 years with autopsy data on 650 participants.[7] High systolic blood pressure (BP ≥160 mm of Hg) in midlife was associated with increased risk of dementia in old age. Hypertension was also associated with lower total brain weight, increased senileplaques (SP) count and hippocampal atrophy. A coexistence of SP and lacunar infarcts further increased the risk of incident dementia. Interestingly, the BP drops around the time of or soon after the onset of clinical dementia. The Kungsholmen project followed 947 people aged ≥75 years every three years for six years.[8] BP decreased significantly over three years prior to and following diagnosis of dementia. In people with baseline systolic BP<160 mm of Hg, a drop of ≥15 mm of Hg over the first follow up was associated with an increased risk of dementia at the second follow up (relative risk, RR=3.1). However, this finding was challenged by another prospective study of 2,356 older people ≥65 years over eight years.[9] In 65-74 years age group high systolic BP and borderline-high diastolic BP was associated with an increased risk of dementia while in those ≥75 years there was a trend towards high systolic BP being associated with low risk of developing dementia. The study concluded that the association between BP and dementia depended on age at which BP was measured and not the time relative to the onset of dementia.

There have been a number of randomized controlled trials (RCT) of anti-hypertensives with cognition and or dementia as one of the outcomes [Table 1]. Some of the earlier trials which included diuretics or beta-blockers were negative but more recent studies have been encouraging. The Syst-Eur trial included 2410 older people with isolated systolic hypertension (systolic BP 160 to 219 and diastolic BP < 95 mm of Hg). The intervention group (*n* =1238) received nitrendipine (calcium channel blocker) and if required, enalapril and hydrocholrthiazide.[10] The intervention group had 50% reduced risk of incident dementia over the two-year follow up. The controls were given antihypertensives at the end of the trial and both groups followed for further two years. The long-term treatment with nitrendipine reduced incident dementia by 55% (from 7.4 to 3.3 cases per 1000 person years).[11] The hypertension in the very elderly trial (HYVET) included patients with hypertension (systolic BP 160-200 and diastolic BP < 110 mm of Hg, respectively) aged 80 years or older who were followed for two years. There was no significant reduction in incident dementia between active (indapamide +/- peridopril) and placebo arms. However, when the data were combined in a meta-analysis with other placebo-controlled trails the combined risk ratio favored active treatment with anti-hypertensive hazard ratio 0.87 (95% CI: 0.76, 1.00).

**Table 1:** Antihypertensive treatment and prevention of cognitive decline: Randomized controlled trials

Randomized controlled trials	Sample size	Participants (characteristics, age in years)	Duration	Treatment	Impact on dementia or cognition
Applegate *et al*. 1994 (systolic hypertension in the elderly)[44]	2034	>60 systolic hypertension	5 years	Chlorthalidone	No significant impact of cognition or incident dementia
Prince *et al*. 1996a (Medical Research Council older people with hypertension)[45]	2584	65-74 yrs; systolic BP: 160-209 mm Hg	4.5 years	Atenolol, hydrochlorothiazide amiloride	No significant impact on cognition or incident dementia
Forette *et al*. 1998 (Syst-Eur)[10]	2418	>60 yrs; systolic hypertension	2 years	Nitrendipine	50% reduction in the incidence of dementia
Forette *et al*. 2002 (Extended follow up of Syst-Eur)[11]	2902	>60 yrs; systolic hypertension	3.9 years	Nitrendipine, enalapril maleate, hydrochlorothiazide	55% reduction in the risk of dementia
Bosch *et al*. 2002 (HOPE)[46]	9297	>55 yrs; left ventricular dysfunction	4.5 years	Ramipril, vitamin E	Significantly better outcome with respect to cognition and function
Lithell *et al*. 2003 (SCOPE)[47]	4964	70-89 yrs; systolic and or diastolic hypertension	3.7 years	Candesartan (antihypertensive used in 84% of controls)	No difference on progression of cognitive impairment
The PROGRESS Collaborative Group (Tzourio *et al* 2003)[48]	6105	Mean age 64 yrs Stroke or TIA with and without hypertension	4 years	Perindopril, indapamide	Significant reduction in cognitive decline and incident dementia associated with recurrent stroke
Peters *et al*. 2008 (HYVET-COG)[49]	3336	Mean age 83 yrs Systolic BP 160-200 and diastolic BP<110 mm Hg	2.2 years	Indapamide peridopril	No significant difference in incident dementia but when data combined with previous trials in meta-analysis results favored active treatment

### Other vascular risk factors

A number of case-control and epidemiological studies have reported hypercholesterolaemia as a risk factor for dementia, including AD. For example, a prospective study with 21 years follow up found raised cholesterol in midlife (≥6.5 mmols/l) to more than double the risk of dementia, including AD.[12] However, not all epidemiological studies show an association between statin use and subsequent dementia.[13] The results of intervention trials, where cognition and dementia are secondary outcomes, have been disappointing.[14,15]

Elevated total homocysteine (tHcy) levels in blood are associated with increased risk of ischaemic heart disease and stroke.[16] In older people, tHcy levels above 15 micromole/L were associated with poor performance on neuropsychological tests and cognitive decline compared to those with levels below 10 micromole/L.[17] In Framingham (USA), 1,092 older people (mean age 76 years) were followed over 8 years. The baseline tHcy levels predicted dementia on follow up. The risk of AD nearly doubled (relative risk, RR 1.8; confidence intervals, CI 1.3-2.5) per one standard deviation increase in tHcy levels at baseline.[18] However, the effect of reducing tHcy on cognition and global functioning has not been adequately investigated. In a small prospective study involving 33 patients, Nilsson *et al*.[19] reported an improvement in cognitive function after two months of cobalamine (B12) and folate treatment in individuals with mild to moderate dementia who had elevated tHcy. Patients with severe dementia and those who had normal tHcy did not however show clinical improvement.

Diabetes is thought to be a risk factor for AD and VaD,[20] especially VaD.[21] Coronary heart disease is associated with increased amounts of cerebral amyloid deposits and increased risk of dementia.[22] A trial fibrillation (even in absence of clinical stroke) was found to have significant positive association with both AD and VaD, and interestingly the association was stronger for AD with cerebrovascular disease than VaD.[23] Smoking has been shown to be risk factor for VaD[24] but the effect of smoking on the risk of developing AD needs further exploration. Some studies find smoking to increase the risk of AD[24–26] while others find no association[27,28] or protective effect.[29,30] The observed protective effect may be due to reduced survival among smokers or the positive effect of nicotine on neuronal survival. So far, there are no RCT which show that treatment of diabetes, heart disease or cessation of smoking prevents incident cases of dementia, AD or VaD.

## POTENTIAL ROLE OF ASYMPTOMATIC CEREBRAL EMBOLI

Above evidence suggests that a number of vascular risk factors may contribute to the clinical syndrome of dementia in AD and VaD. However, underlying pathophysiological mechanisms need further exploration, especially if there are any common pathways by which different risk factors eventually lead to cerebral damage. Stroke or transient ischaemic attacks (TIA) may be one such common pathway. Over recent years, there has been growing interest in the role of asymptomatic spontaneous cerebral emboli (SCE) in the causation of progressive brain damage and dementia. Such micro-emboli have been shown to be frequent in patients with severe carotid artery disease, valvular heart disease, and stroke.[31,32] In these patient groups and in those undergoing heart bypass surgery cerebral emboli have been shown to predict future risk of cerebrovascular accidents and poor neurocognitive outcomes.[33,34]

In Manchester (UK), we conducted a case-control study which included 85 patients with AD, 85 patients with VaD and 150 age and sex matched controls. In just one hour of transcranial Doppler monitoring SCE were detected in middle cerebral arteries in 32 (40%) AD and 31 (37%) VaD patients compared to 12 (15%) and 12 (14%) of their respective controls. The odds ratio for the presence of SCE was 2.70 (1.18 - 6.21) for AD and 5.36 (1.24 - 23.18) for VaD.[35] In controls, the presence of SCE was associated with cardiovascular risk factors (a history of stoke or transient ischemic attack, a history myocardial infarction or angina, higher diastolic blood pressure, presence of severe carotid artery stenosis) and current treatment with anti-platelet medications. However, carotid stenosis and other vascular risk factors did not explain the increased frequencies of SCE in patients with dementia. It may be that some other factors that increase propensity for embolus formation are involved. Abnormalities in coagulation pathways and platelet activation have been reported in patients with AD.[36,37] We investigated the clinical relevance of SCE by conducting a longitudinal follow up of patients with dementia over six months.[38] A total of 132 patients had validated SCE assessment and at least one of the outcome measure data initially and at six months. Patients with dementia who were SCE positive (*n* =47, 36%) at initial assessment showed a statistically significant more rapid decline in cognitive functioning and activities of daily living over six months compared to SCE negative patients. These results were unaltered after adjusting for ApoE4 status and the use of cholinesterase inhibitors and or antiplatelet drugs.

### SCE and potential mechanisms of brain damage in dementia

The mechanism by which cerebral micro-emboli cause brain damage is not known but is presumably by microischaemic changes. We did not find SCE to be associated with either infarcts or severity of white matter hyperintensities (WMH). However, one of the mechanisms of cerebral embolization (venous to arterial circulation shunt suggestive of patent foramen ovale in the heart) was associated with increased severity of deep and peri-ventricular WMH in patients with AD alone.[39] However, it is likely that SCE do not lead to a structural evidence of cerebrovascular disease that is large enough to be detected by the currently available neuroimaging techniques. The initial vascular insult resulting from embolization of microvessels may trigger another mechanism of brain damage, such as inflammation, but not leave any evidence of the original vascular insult. In AD, cerebral microvessels have been shown to release significantly higher amounts of inflammatory mediators such as interleukin-1β, IL-6, and tumor necrosis factor α.[40] It is also proposed that the microglias in the diseased or aged brain are ‘primed’, and switch their phenotype to produce neurotoxic molecules when they respond to systemic inflammatory signals.[41] Systemic infections are suggested as potential triggers for microglial activation. However, microglia are sensitive to other disturbances of brain homeostasis[42] that may include SCE induced ischemia in cerebral microcirculation.

## PROBLEMS FOR FUTURE RESEARCH

The current literature on the control of vascular risk factors in prevention of dementia has certain limitations. Most RCTs target hypertension with a few targeting cholesterol. None of the RCTs attempt to optimize control of multiple vascular risk factors. Cognition and dementia is a secondary outcome with limited statistical power to detect significance of any true difference between intervention and placebo groups due to low frequencies of incident dementia in the selected population. There are not any prevention trials (except for atorvastatin) targeting specifically people at most risk of developing dementia. The term mild cognitive impairment (MCI) is commonly used to describe a group of patients who have some cognitive deficits but not severe enough to affect daily functioning and warrant a diagnosis of dementia.[43] However, tests used to assess cognition and daily functions and diagnostic criteria for MCI vary. The trials targeting older people with MCI with incident dementia as the primary outcome are likely to need at least 2000-3000 participants followed for two to three years. It is essential to develop surrogate markers to help reduce the size of the study and associated costs. We do not know whether there is a therapeutic time window between midlife and late life during which vascular risk factors need to be controlled to achieve a reduction in the risk of dementia. Also, lower the better doctrine applied to BP and cholesterol in cardiovascular and stroke prevention trails may not necessarily hold true for the outcome of cognition in people with MCI. Lastly, we need RCTs that optimize the control of multiple risk factors as co-morbidity is common in older people.

## CONCLUSIONS

Over the next few decades, dementia is going to be a major health problem worldwide, especially in developing countries like India. Alzheimer’s disease (AD) and VaD are two main causes with mixed dementia being common. Epidemiological evidence has shown that a number of vascular risk factors in midlife are associated with dementia in late life. Randomized controlled trials (RCT) evidence that treatment of vascular risk factor prevents dementia exists for hypertension but not for others. RCT studies are limited in their methodology. Dementia is always a secondary outcome and studies do not specifically focus on population at highest risk of dementia (for example; those with mild cognitive impairment). Asymptomatic cerebral emboli may be a novel mechanism of vascular brain damage that warrants further exploration. The interventions to inhibit emboli formation are already available. We are currently conducting a pilot study investigating two such therapies (clopidogrel and atorvastatin) in patients with dementia.
